# Supplementary material for: ROS-induced ATP synthase mRNA degradation and metabolism dysfunction reveals the mechanism of artificial deteriorated cotton seeds
Source: PLoS One. 2026 Feb 10;21(2):e0339977. doi: 10.1371/journal.pone.0339977 (PMC12890139; doi:10.1371/journal.pone.0339977)
Supplement: S1 Table — (PDF) [file pone.0339977.s001.pdf]

Double primers used for gene integrity detection

ATP synthase subunit	Accession number	Forward/Reverse primes sequence (5'-3')	Primer length (bp)	Annealing temperature (℃)	Product size (bp)
α	*XM_016819324-5'F*	GCACTGGATCTATTGTGG	18	58.9	108
	XM_016819324-5'R	GTTCGTGATCGCTTAGAG	18	59	
	XM_016819324-3'F	AACACGAACTCTATCGGA	18	59.1	105
	XM_016819324-3'R	TGTGCCTTTCTATTAAGCC	19	59	
β	*XM_016824591-5'F*	AAGATCACTGATGAGTTCAC	20	58.9	199
	XM_016824591-5'R	CATCCATAGCAATGGTCC	18	59	
	XM_016824591-3'F	CGAGCTTAGTGAAGATGATAA	21	59.1	128
	XM_016824591-3'R	TCTCCTTCAACTCCACAT	18	58.9	
γ	*XM_016875018-5'F*	TGATACACGAACAACAGC	18	59.1	187
	XM_016875018-5'R	ATATAACGAACTCCAATAGGAC	22	59	
	XM_016875018-3'F	TGTTCGGACTTGTTCATG	18	59	136
	XM_016875018-3'R	TACCAACTTATGCTGATGTC	20	59	
ε	*XM_016862594-5'F*	ACAACACAGCCAAAGAAA	18	59.1	97
	XM_016862594-5'R	GCATATGTTCGAGTAGGTTAT	21	59.1	
	XM_016862594-3'F	ATGATGTTGCACATCCTAAT	20	59	91
	XM_016862594-3'R	ACAAGAATACTTGATAACCCAT	22	59.1	
δ	*XM_016861180-5'F*	AACATGGACCCTCCTAAA	18	58.9	88
	XM_016861180-5'R	AATTGACAGTAAGCTTGGAA	20	59.1	
	XM_016861180-3'F	CTTAAGATTCATATGATGGATGC	23	59.1	198
	XM_016861180-3'R	TTGAACTGCAATAAGAACAAC	21	59.1	
Actin	*NM_001155179.1-5' F*	GTATGAGCAAGGAGATCAC	19	58.9	194
	NM_001155179.1-3 'R	TTAGAAGCACTTCATGTGG	19	59	
