# Supplementary material for: ROS-induced ATP synthase mRNA degradation and metabolism dysfunction reveals the mechanism of artificial deteriorated cotton seeds
Source: PLoS One. 2026 Feb 10;21(2):e0339977. doi: 10.1371/journal.pone.0339977 (PMC12890139; doi:10.1371/journal.pone.0339977)
Supplement: S2 Table — (PDF) [file pone.0339977.s002.pdf]

Relative expression levels of the 5' and 3' ends of each subunit

ATP synthase subunit		Relative Expression		Standard Deviation	
		5’ end	3’ end	5’ end	3’ end
α	CK	1.00	1.02	0.11	0.21
	3d	2.53	0.81	0.64	0.23
β	CK	1.02	1.00	0.23	0.08
	3d	0.84	0.99	0.10	0.10
γ	CK	1.07	1.00	0.43	0.05
	3d	0.26	5.27	0.02	2.87
ε	CK	1.00	1.01	0.11	0.13
	3d	0.84	1.03	0.45	0.38
δ	CK	1.02	1.03	0.22	0.28
	3d	0.77	2.42	0.29	1.46
