# Supplementary material for: ROS-induced ATP synthase mRNA degradation and metabolism dysfunction reveals the mechanism of artificial deteriorated cotton seeds
Source: PLoS One. 2026 Feb 10;21(2):e0339977. doi: 10.1371/journal.pone.0339977 (PMC12890139; doi:10.1371/journal.pone.0339977)
Supplement: S3 Table — (PDF) [file pone.0339977.s003.pdf]

R value

ATP synthase subunit	R value		Standard Deviation	
	CK	3 d	CK	3 d
α	1.00	0.32	0.08	0.02
β	1.00	1.18	0.09	0.06
γ	1.00	0.24	0.05	0.02
ε	1.00	0.88	0.10	0.02
δ	1.00	0.80	0.05	0.04
