# Supplementary material for: ROS-induced ATP synthase mRNA degradation and metabolism dysfunction reveals the mechanism of artificial deteriorated cotton seeds
Source: PLoS One. 2026 Feb 10;21(2):e0339977. doi: 10.1371/journal.pone.0339977 (PMC12890139; doi:10.1371/journal.pone.0339977)
Supplement: Attachment — Submitted filename: Response_to_Reviewers.docx [file pone.0339977.s006.docx]

Response to Reviewers

Manuscript title: ROS production and loss of ATP synthase subunit mRNA integrity as a mechanism of artificial deteriorated cotton seeds

Journal: PLOS ONE

Authors: Ci Song, Junming Zhang, Zhenzhen Xing, Fengxu Gu, Junying Chen*

We sincerely thank the editor and reviewers for their careful reading and constructive comments. We have carefully revised our manuscript according to all suggestions. Below, we provide detailed responses point by point, with modifications highlighted in the revised manuscript and summarized with artificial line numbers.

**Response to Reviewer 1**

Comment 1:

Can you please reconsider the title with a unique word as it seems so simple and less attractive than the audience.

Response:

Thank you for your valuable suggestion. We agree that a more specific and engaging title will help attract readers’ attention. We have revised the title to better emphasize the mechanistic focus and novelty of our findings.

Modification:

Before (Title-L1): ROS production and loss of ATP synthase subunit mRNA integrity as a mechanism of artificial deteriorated cotton seeds.

After: ROS-induced ATP synthase mRNA degradation and metabolism dysfunction reveals the mechanism of artificial deteriorated cotton seeds



Comment 2:

Abstract and conclusion are too simple; no novelty or significance is highlighted.

Response:

We appreciate the reviewer’s advice. We have rewritten the abstract and conclusion to highlight the novelty, emphasizing the mechanistic link between ROS accumulation and ATP synthase mRNA integrity loss, and its implications for seed vigor and germplasm preservation.



Comment 3:

Please double-check grammar; overall language needs verification.

Response:

We have carefully revised the entire manuscript for grammar, tense consistency, and scientific style.

Comment 4:

Please rewrite the introduction or add constructive information to make it attractive.

Response:

We have enriched the Introduction by emphasizing the gap in understanding mRNA oxidation during cotton seed aging.

Comment 5:

Add graphical/pictorial representation of your work along the abstract.

Response:

We have prepared a Graphical Abstract illustrating the sequence: ROS accumulation → mRNA oxidative damage → impaired ATP synthesis → Mitochondrial dysfunction. The figure provides an intuitive summary of our findings.

(A figure are provided below this document.)

Comment 6:

Graphs are too small and fonts unclear.

Response:

All figures have been resized to improve readability(Results section, Figs. 1–10).

Comment 7:

Add more justification in discussion and include relevant new findings.

Response:

We have expanded the Discussion to better connect ROS accumulation, mitochondrial dysfunction, and ATP synthase mRNA damage. We also referenced recent literature supporting the role of mRNA oxidation in post-transcriptional regulation during seed aging.

Comment 8:

Ensure references follow journal pattern.

Response:

All references have been reformatted according to PLOS ONE guidelines, including DOI addition and consistent author-year style.

**Response to Reviewer 2**

Comment 1:

Explain why cultivar “Xinluzao 74” was chosen.

Response:

We have clarified this in Introduction “Xinluzao 74” is a major high-yield, disease-resistant cultivar widely cultivated in Xinjiang, representing typical seed storage behavior of upland cotton. Its characteristics make it an ideal model for artificial aging studies with global relevance.

Comment 2:

Justify 45°C and 100% RH conditions.

Response:

We have added in Method part:

“These conditions follow Tesnier et al. (2002) and are widely used to simulate accelerated seed aging, providing reproducible deterioration kinetics comparable across oilseed species.”

Comment 3:

Expand figure legends for stand-alone clarity.

Response:

Revised all figure legends (Figs. 1–10) to include species, treatment, replicates, and statistical test information, ensuring they can be understood independently.

Comment 4:

Quantify TTC staining intensity.

Response:

We appreciate the reviewer's suggestion to quantify the TTC staining intensity. While we agree that quantification can be valuable, the visual difference in staining between the experimental and control groups is consistently clear and striking across all replicates, as shown in Figure 2. Furthermore, we would like to highlight that the dehydrogenase activity assay (presented in Figure 3 provides a precise and quantitative measurement of the same biological process that the TTC staining visually represents. We believe that this enzymatic data robustly supports our qualitative TTC observations.

Comment 5:

Discuss conductivity—possible non-aging effects.

Response:

While conductivity increase mainly reflects membrane leakage from aging, minor variation due to seed coat permeability cannot be excluded and warrants further study, so we have added clarification in case any possible misunderstanding.

Comment 6:

Interpret initial H₂O₂ decline more carefully.

Response:

The transient drop in H₂O₂ after 5 days might also result from peroxidase-mediated conversion or ROS-induced degradation, rather than exclusive scavenging, so we have added clarification in the Discussion part.

Comment 7:

Clarify whether ATP synthase and respiration decline are cause or consequence of mRNA damage.

Response:

The observed reduction in ATP synthase activity is likely a downstream effect of mRNA integrity loss, suggesting that ROS-induced mRNA oxidation may precede metabolic inhibition, so we added mechanistic clarification in Discussion part:

Comment 8:

Improve discussion—avoid repetition.

Response:

We revised the first two Discussion paragraphs to merge redundant result descriptions and focus on mechanistic interpretation and broader biological implications.

Comment 9:

Minor English issues.

Response:

Thanks for your comments, we have rephrased all informal expressions.
